# The crisis of manufactured scholarship: confronting AI-driven “letter-bombing” and profile inflation in medical journals

**DOI:** 10.1186/s12957-026-04465-6

**Published:** 2026-06-24

**Authors:** Manoj Pandey

**Affiliations:** 1 Department of Surgical Oncology, Institute of Medical Sciences, Banaras Hindu University, Varanasi, 221005 India; 2 DHR-ICMR Advanced Molecular Oncology Diagnostic Services (DIAMOnDS), Institute of Medical Sciences, Banaras Hindu University, Varanasi, 221005 India; 3 Health Technology Assessment India (HTAIn) resource center, Institute of Medical Sciences, Banaras Hindu University, Varanasi, 221005 India

**Keywords:** Publication Ethics, Medical Publishing, Generative Artificial Intelligence, Large Language Models (LLMs), Scientific Correspondence, Surgical Oncology, Bibliometrics, Peer-Review Integrity, Academic Misconduct

## Abstract

Post-publication peer review faces a emerging systemic vulnerability from ‘letter-bombing’—the proliferation of high-volume, potentially machine-generated critiques that exploit citable correspondence units, creating a plausible risk of academic metric inflation. Driven by the democratization of Large Language Models (LLMs), these automated submissions frequently exploit formatting loopholes to manipulate public indicators like the h-index. The problem is particularly observable in data-intensive specialties like surgical oncology, where abstract-level statistical critiques targeting complex clinical trials risk muddying translational research communication. Drawing on documented bibliometric anomalies and confirmed editorial actions, this piece discusses operational definitions of abnormal correspondence volumes and proposes a multi-layered framework. To protect the scientific record, we propose an enforceable framework for editorial boards: implementing strict metadata labeling to close indexing loopholes, verifying author history, and mandating the “Right of Simultaneous Reply” as a universal baseline publishing standard.

**Graphical Abstract:**

**Figure:**
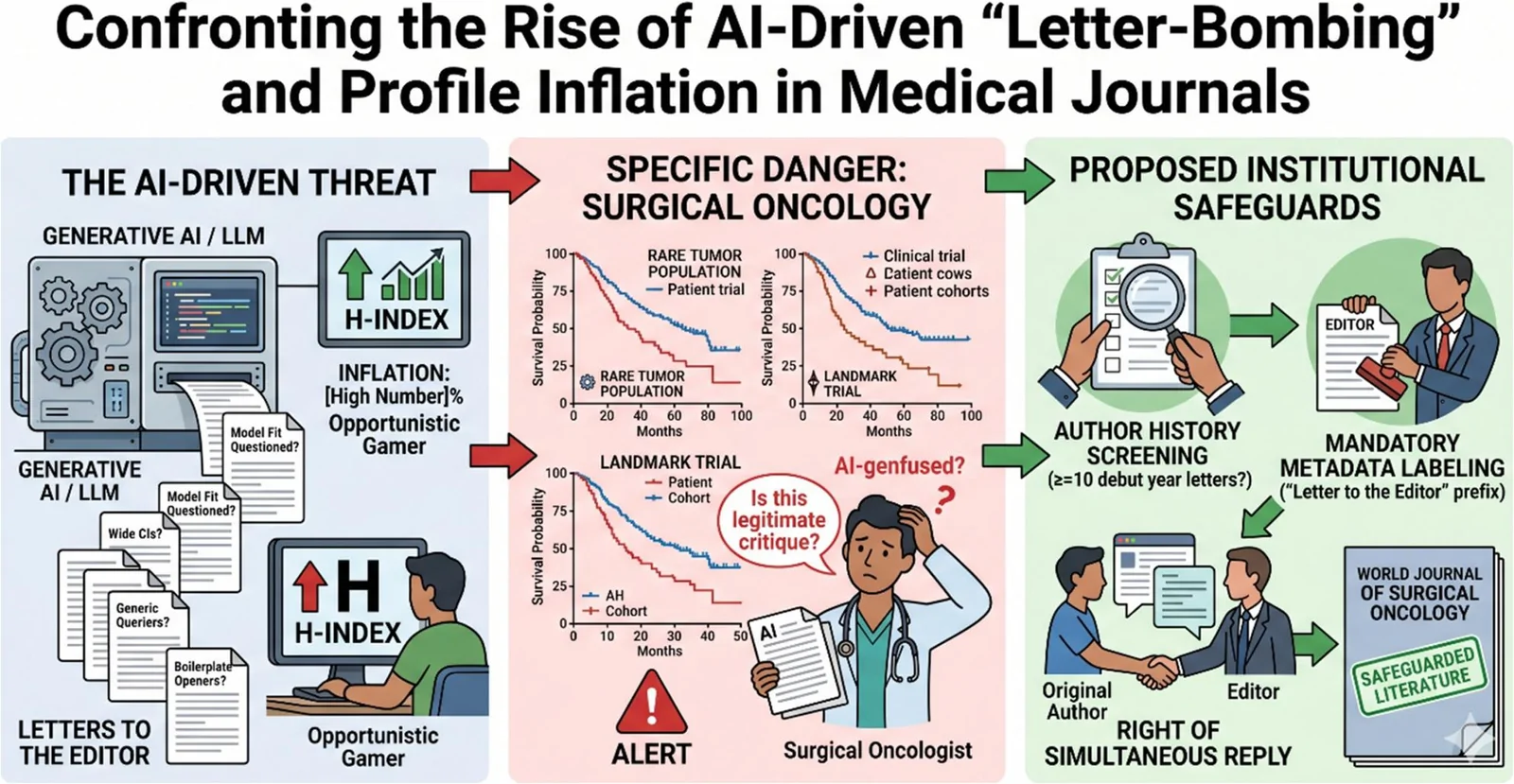

## Introduction

Post-publication peer review is built on a straightforward premise: published data must remain open to expert scrutiny, correction, and debate. Historically, letters to the editor and formal commentaries served as a vital safety net where readers shared clinical insights, exposed methodological flaws, and refined medical evidence. As Kumar (2022) [1] emphasized in his examination of the journal reader’s role, letters represent a critical scientific contribution, enabling direct dialogue that strengthens the medical literature. When a reader invested the time to analyze primary data and write a formal commentary, it signaled authentic academic engagement. Even among medical trainees, quantitative evidence shows that drafting correspondence has long served as a legitimate tool for developing critical thinking and early professional identity [2].

**Fig. 1:**
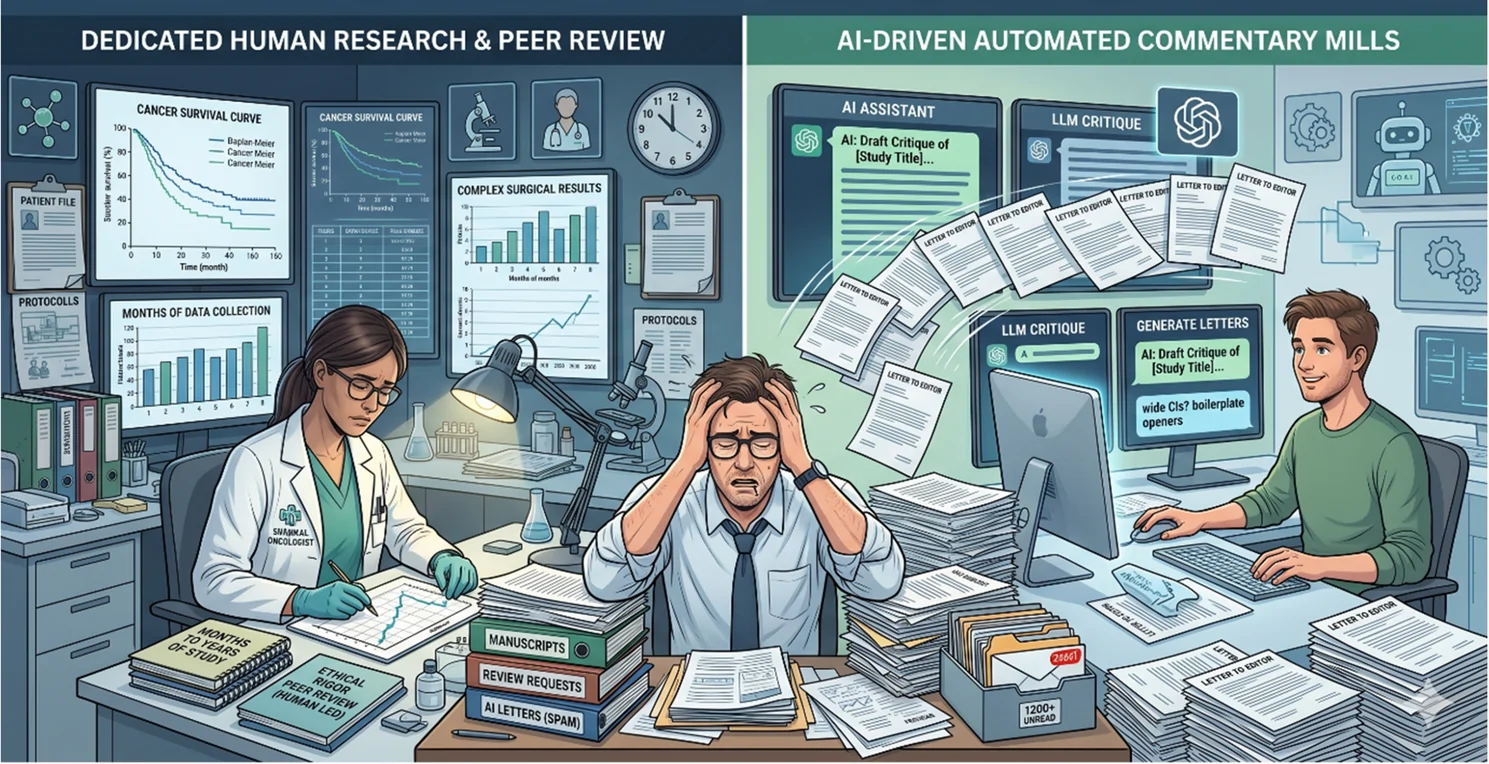
The modern imbalance in scholarly exchange

Today, this tradition is being actively undermined by a metric-gaming tactic known as “systematic correspondence” or “letter-bombing”. Rather than advancing science, coordinated groups and individual actors flood journals with high volumes of critiques spanning an implausibly diverse range of medical specialties. Generative artificial intelligence (AI) has supercharged this practice, turning what was once a labor-intensive manual effort into a cheap, automated production line that exploits publication metrics (Fig. 1).

This volume-driven approach causes immediate, distinct friction in data-heavy specialties like surgical oncology. Landmark oncological advances frequently rely on highly specific clinical trial designs, non-randomized real-world evidence, and niche patient sub-cohorts. In rare tumor populations, sample sizes are inherently limited and variables are tightly controlled. When automated Large Language Models (LLMs) subject these complex, multi-modal surgical studies to superficial, abstract-level statistical nitpicking, the resulting critiques are fundamentally decoupled from clinical reality. Blindly casting doubt on survival metrics, risk-prediction models, margin-status analyses, or adjuvant protocols without understanding the underlying surgical and biological realities does not improve science; it simply confuses clinical readers and stalls translational oncology research.

### The scale of the modern crisis

This surge in manufactured critiques is a predictable byproduct of a persistent “publish or perish” culture, now accelerated by easy access to LLMs. Recent data reveals the alarming scale of this integrity crisis. Between 2023 and 2025, an unusual cohort of “prolific debutante” authors; individuals with no prior publication record in scientific correspondence suddenly emerged in the top 5% of letter writers globally. As documented in *Science*, analyses found a 376% increase in authors publishing ten or more letters during their debut year immediately following the mainstream adoption of generative AI tools [3]. This behavior builds on metric-driven incentive structures that reward publication volume over actual substance, a systemic flaw that AI tools have scaled exponentially [4].

In extreme scenarios, single authors have been found to produce dozens or hundreds of letters within a few months, shifting effortlessly between unrelated fields as disparate as tropical medicine and neurosurgery. Editors at major journals report receiving near-identical, template-driven critiques within days of a study’s publication. *The New York Times* highlighted a case where a suspicious, potentially AI-generated persona targeted multiple editors simultaneously with polished but highly formulaic submissions [5]. While AI-detection tools frequently flag these pieces for repetitive linguistic patterns and factual inaccuracies, the sheer volume continues to overwhelm the gates of scholarly publishing.

Further investigations by McGill’s Office of Science and Society revealed extreme outliers: one physician published zero letters in 2024 but unleashed 84 across 58 unrelated topics in 2025, while another produced 234 letters in a single year after a completely blank historical baseline [6]. These patterns leave little doubt regarding automation and expose the implausibility of true multi-specialty expertise.

### The anatomy of a “letter-bomb”

**Fig. 2:**
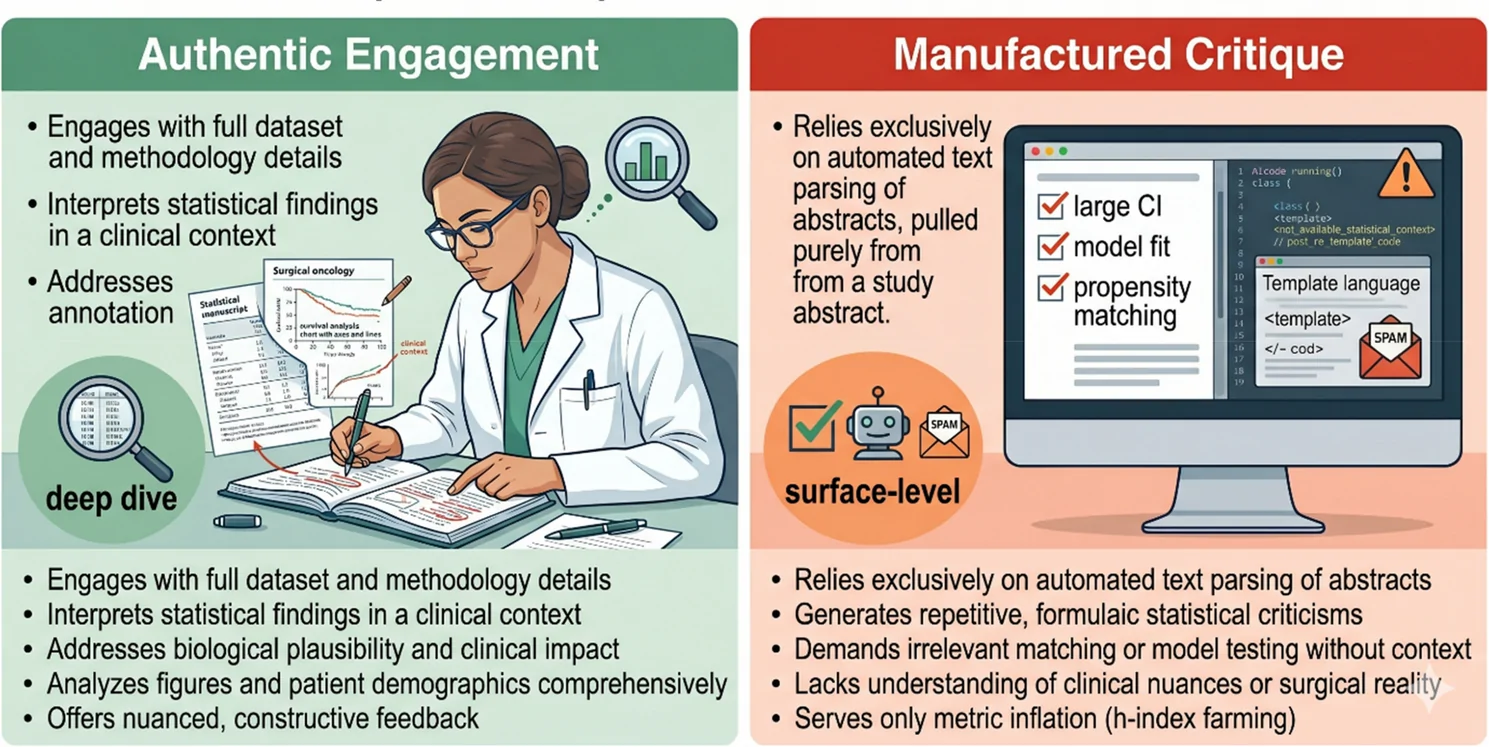
Genuine expert critique vs. AI-generated letter-bomb (manufactured critique)

Manufactured correspondence is characterized by a systematic approach that shares a predictable and generic DNA (Fig. 2). The process typically involves pasting a published abstract or full text into an LLM with instructions to find faults or generate statistical nitpicking. Comprehensive preprint analyses titled “Robot Pen Pals” have documented this trend, mapping how these automated loops rely on recycled, formulaic arguments that lack clinical or contextual depth [7].

These letters focus heavily on generic statistical critiques. They question model fit, point out wide confidence intervals, or demand propensity-score matching as a default reflex, completely ignoring whether these points are relevant to the study’s specific design or constraints. For example, criticizing model fit in a purely descriptive behavioral study [8, 9], or complaining about wide confidence intervals in a niche patient subpopulation where sample sizes are naturally constrained, demonstrates zero engagement with the actual research context.

Instead of addressing real data, these letters rely on highly polished, scholarly sounding language. This allows a single operator to generate and submit dozens of critiques a week with minimal effort, wasting the time of editorial teams and original authors without adding any value to the field. A clear real-world example occurred when an AI-generated letter targeted a *New England Journal of Medicine* paper on malaria [6]. The automated critique falsely accused the authors of overlooking their own prior work on mosquito resistance, illustrating how easily these tools fabricate scientific issues without substantive engagement.

### Profile of the serial commenter

The modern serial commenter represents a highly calculated evolution in academic gaming. These individuals maintain publication portfolios that defy professional and temporal probability, claiming overnight expertise in fields ranging from urology and nuclear cardiology to ophthalmology and community medicine. Their strategy relies entirely on volume: using automated alerts to scan PubMed, scraping abstracts, and dispatching mass critiques without ever reading beyond the abstract. While a *Retraction Watch* investigation previously profiled a pre-AI author who published over 500 letters in a single year, LLMs have now commoditized and scaled this metric manipulation strategy [10].

To move past purely rhetorical descriptions, editorial boards need clear, operationalized bibliometric thresholds to identify “letter-bombing”. Based on established frameworks for extreme publishing behavior [4], we define abnormally prolific correspondence using a composite indicator: The publication of > 10 letters to the editor within an author’s debut year in scientific correspondence and a career trajectory where an author’s correspondence outputs outnumber their original peer-reviewed datasets by a ratio greater than 5:1.

However, editorial boards must treat these quantitative thresholds strictly as preliminary screening flags rather than definitive evidence of misconduct, establishing rigorous safeguards against false positives. Legitimate biostatisticians, dedicated clinical methodologists, and early-career trainees frequently publish high volumes of correspondence as an essential component of scientific oversight. To protect these vital voices, an automated flag must merely prompt a secondary, qualitative editorial review. If the correspondence demonstrates granular engagement with the full text (rather than generic, abstract-level text scraping), introduces unique re-analyses of data, or is authored by individuals with verifiable domain training, the flag should be dismissed. Quantitative filters must protect the journal’s capacity, never suppress authentic, rigorous academic debate. Public records have begun to expose the extent of this practice [6] e.g. a physician who published zero letters in 2024 unleashed 84 letters across 58 unrelated topics in 2025, a pattern that prompted immediate suspicion when AI-detection tools scored the submissions as machine-generated. Another author reportedly produced over 234 letters in 2024, as noted in the same analysis.

The underlying motivation is transparent: each published letter counts as a citable output in indexing systems that reward unweighted h-index growth for promotion, funding, or international mobility. Yet, they contribute no new findings and do nothing to improve patient care; they are metric manipulation dressed in the language of critique. It is, as described in *Science*, a quick and dirty route to higher impact numbers [11].

### The burden on the scientific record

Allowing unchecked, AI-generated correspondence to flood medical literature creates profound systemic risks:

- *Editorial Overload*: Small editorial teams are forced to expend limited operational resources vetting, tracking, and coordinating rebuttals for low-quality submissions, diverting valuable time away from primary research that directly shapes clinical guidelines.
- *Reader Misclassification*: A polished, machine-generated commentary appearing without a simultaneous response can cast unwarranted doubt on solid research. Clinical readers and trainees often accept these critiques at face value, unaware of their automated origins.
- *Devaluation of Genuine Labor*: Researchers spend years securing ethical approvals and collecting primary patient data, only to see their work targeted by a superficial critique generated in seconds. When an academic profile consists of hundreds of generic letters rather than primary data, it fundamentally questions the credibility of the broader literature.

Medical journals across specialties have issued urgent warnings regarding this rapid infiltration of undeclared AI content into the commentary literature [12, 13].

## Documented evidence and systemic risks

The threat has moved past theoretical concern. In early 2025, *Neurosurgical Review* retracted 129 letters and commentaries suspected of mass production via undeclared AI tools, marking one of the largest structural integrity cleanups to date. The overwhelming volume and quality issues eventually forced the journal to temporarily pause all correspondence submissions to protect its editorial capacity [14]. Similar disruptions have hit multiple medical specialties, pushing editors to adopt stricter verification protocols and AI-screening tools.

Ethical frameworks explicitly state that letters to the editor should not be generated by AI, citing clear violations of authorship responsibility and practical harms to literature tracking [15]. When the academic community can no longer distinguish expert analysis from automated noise, trust in the post-publication review process breaks down.

Patient care can suffer indirectly if flawed commentaries create hesitation around valid clinical therapies, and the practice actively worsens global academic inequities. Researchers focused on genuine, resource-intensive clinical discovery are forced to compete for visibility against metric-gamers exploiting automated workflows. In environments where publication pressure is high but local resources are limited, the temptation to adopt these factory-style tactics threatens the integrity of regional medical journals. AI policies at major journals have largely failed to curb this surge, leaving systemic vulnerabilities unaddressed [16, 17].

## Remedies: safeguards and accountability

Defending the scientific record requires a coordinated shift in editorial policy, aligned with core guidelines from the Committee on Publication Ethics (COPE) and the International Committee of Medical Journal Editors (ICMJE, 2026) [18].

The primary structural remedy is universalizing the “Right of Simultaneous Reply”. Top-tier journals have shown that a letter criticizing a study should only be published alongside a direct response from the original research authors; this must become the industry baseline for all formal correspondence [19, 20]. Publishing the critique and the rebuttal concurrently in the same issue maintains a balanced perspective for the reader and acts as a natural deterrent against automated attacks. An opportunistic author is far less likely to submit a generic, machine-generated critique if they know it will be instantly exposed and answered by the experts who conducted the primary study.

**Fig. 3:**
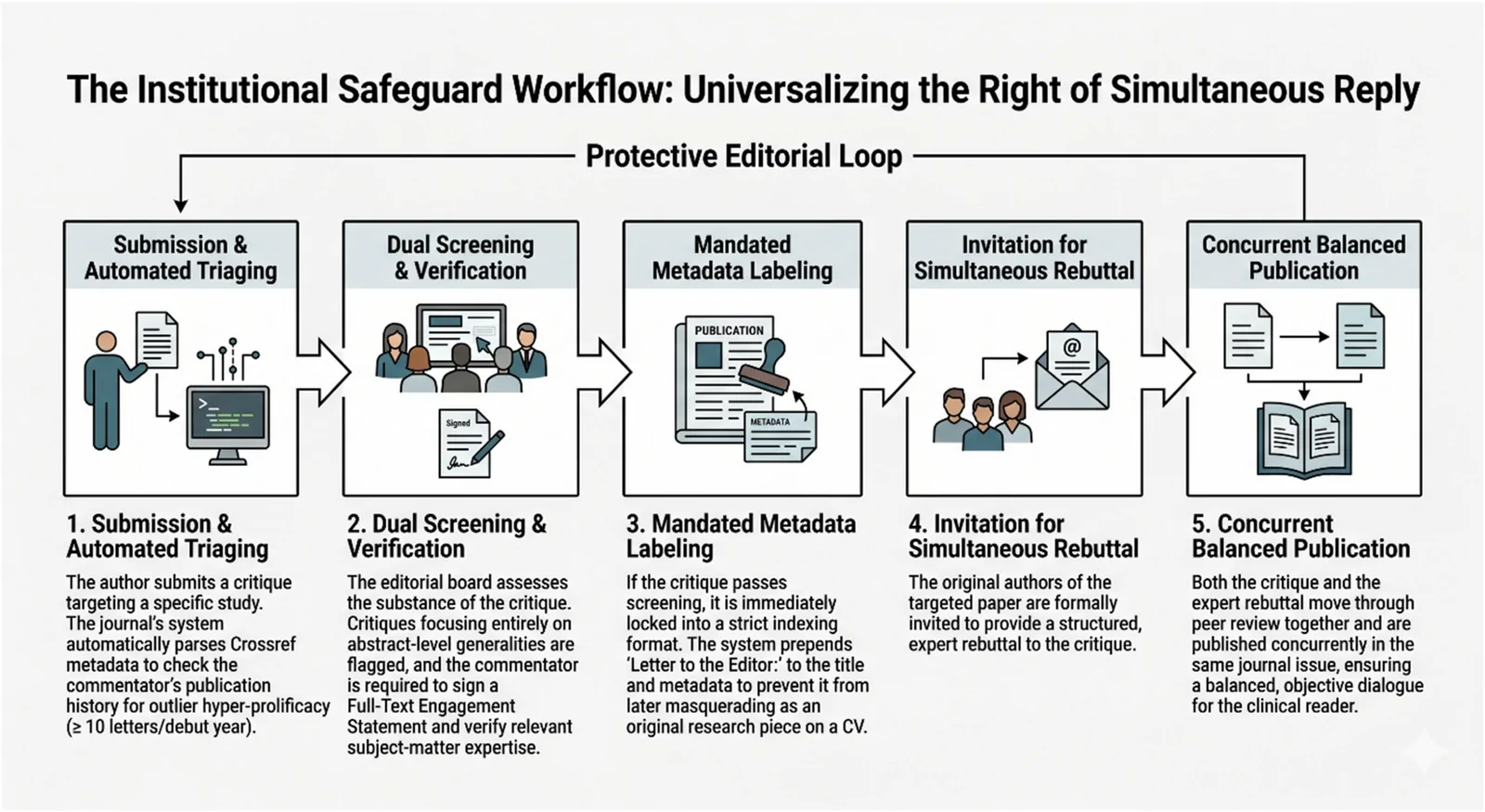
Institutional safe guard workflow: ensuring balanced scholarly dialogue

Furthermore, editorial boards should enforce the following operational safeguards (Fig. 3):

- *Initial Submission Screening*: ScholarOne, Editorial Manager, or proprietary submission portals can ingest the corresponding author’s ORCID iD to auto-populate historical publication counts. If the user hits the ≥ 10 letters/year threshold, the system flags the manuscript for the handling editor.
- *Qualitative Evaluation*: The handling editor evaluates if the critique addresses specific clinical trial nuances or relies on generic LLM-style prompt templates (e.g., repetitive critiques of sample size or confidence intervals). Cross-reference incoming submissions against the author’s history to flag individuals displaying outlier hyper-prolificacy metrics [3, 7].
- *AI Detection and Template Checks*: Critically evaluate correspondence that focuses exclusively on abstract-level information or matches known LLM prompt structures [12, 16].
- *Expertise Verification*: Require that commentators demonstrate clear, documented expertise or a relevant publication footprint in the specific clinical area they are critiquing.
- *Full-Text Engagement Statements*: Mandate a formal declaration confirming the authors have reviewed the full manuscript, figures, and supplementary datasets, rather than just the abstract text.
- *Standardized Crossref Metadata Mapping*: To prevent letters from masquerading as primary datasets during automated database scrapings, publishers must mandate that all correspondence metadata explicitly feature the type="letter” or type="commentary” attribute within their XML schema uploads to Crossref and PubMed Central. This ensures that public tracking tools do not aggregate short correspondence units into primary citation weights.
- *Reporting Misconduct*: Report clear instances of manufactured or undeclared AI correspondence to institutional deans or research directors, maintaining compliance with ethical oversight guidelines (ICMJE, 2026) [18].

*Clinical Orthopaedics and Related Research’s* proactive approach serves as an operational model for managing AI-assisted letters [14, 19]. To combat the erosion of academic integrity, we propose a standardized workflow where critiques and author rebuttals are published concurrently to ensure a balanced scholarly dialogue.

### Operational realities: infrastructure bottlenecks at WJSO

To see how these structural vulnerabilities play out in daily practice, it is instructive to look at the operational front lines of the *World Journal of Surgical Oncology* (WJSO). At WJSO, the volume of accepted and published correspondence is kept strictly controlled and exceedingly small. We do not treat letters as secondary citable units; every single submission undergoes the exact same rigorous peer-review workflow applied to primary articles.

The real bottleneck is not editorial resolve, but the legacy architecture of current manuscript submission software. Standard platforms lack the native infrastructure to handle coordinated correspondence workflows. The system does not allow an incoming letter to be digitally locked to the original target article’s DOI, nor does it feature an automated pipeline to invite, track, and combine the original authors’ rebuttals into a single peer-review thread.

Instead, this linkage must be handled manually through reference strings and post-production Crossref metadata mapping. The downstream result of these fragmented, manual tracking workflows is a clear trend toward misclassification in public repositories. An audit of historical indexing records in databases like PubMed shows a persistent metadata vulnerability: letters are frequently indexed ambiguously, allowing them to masquerade as full, primary empirical datasets during automated database scrapings.

To solve this, publishers must mandate structural modifications to electronic submission infrastructure. The submission portal should require commentators to input the exact DOI of the targeted article upon initial upload. This step must trigger an automated “parent-child” digital relationship in the system metadata, preventing misclassification from day one and automatically sending a peer-review invitation to the original corresponding authors. Without these automated software-level upgrades, small editorial teams will continue to face an asymmetrical administrative burden trying to stop automated metric gaming with manual oversight.

## Conclusion: reclaiming scholarly integrity

The core mission of medical publishing remains the dissemination of verified truths to improve clinical care. Treating the scientific record as a playground for profile-building undermines this goal and devalues the collective efforts of the global research community. While authentic, expert-driven debate remains the lifeblood of medical progress; historically proven by landmark correspondence that advanced fields like DNA structure, *Helicobacter pylori*, and thalidomide safety, the community must condemn the practice of manufactured scholarship.

By prioritizing quality over quantity, implementing mandatory simultaneous reply workflows, and leveraging software-level safeguards, journals can protect scholarly discourse. Addressing automated letter-bombing through clear policy and modern submission tools will ensure that post-publication review fulfills its original promise: functioning as a rigorous arena for advancing medical science rather than a factory for unearned metrics.

## Data Availability

No new datasets were generated or analyzed during the preparation of this manuscript.
